# Editorial: Neuromodulation technology: advances in optics and acoustics

**DOI:** 10.3389/fncel.2024.1494457

**Published:** 2024-10-03

**Authors:** Meijun Ye, Chen Yang, Ji-Xin Cheng, Hyeon Jeong Lee, Ying Jiang, Linli Shi

**Affiliations:** ^1^Division of Biomedical Physics, Office of Science and Engineering Laboratories, Center for Devices and Radiological Health, Food and Drug Administration, Silver Spring, MD, United States; ^2^Office of Neurology and Physical Medicine, Office of Product Evaluation and Qualification, Center for Devices and Radiological Health, Food and Drug Administration, Silver Spring, MD, United States; ^3^Department of Chemistry, Boston University, Boston, MA, United States; ^4^Department of Electrical and Computer Engineering, Boston University, Boston, MA, United States; ^5^Department of Biomedical Engineering, Boston University, Boston, MA, United States; ^6^College of Biomedical Engineering & Instrument Science, Key Laboratory for Biomedical Engineering of Ministry of Education, Zhejiang University, Hangzhou, Zhejiang, China; ^7^MOE Frontier Science Center for Brain Science & Brain-Machine Integration, Zhejiang University, Hangzhou, Zhejiang, China; ^8^Department of Biological Engineering, Massachusetts Institute of Technology, Cambridge, MA, United States

**Keywords:** neuromodulation, photovoltaic, optogenetic, sonogenetic, ultrasound

Neuromodulation technologies, which interface directly with the nervous system, promise transformative advances in treating neurological disorders and resolving fundamental neuroscience questions. In addition to conventional electrical stimulation, recent innovations in optical and acoustic methods are reshaping basic neuroscience research and opening the clinically relevant therapeutic possibilities by offering minimally invasive neuromodulation with higher spatial and/or temporal resolution. This Research Topic collection assembles recent articles reporting novel optical and acoustic neuromodulation tools and their new applications.

## Optical innovations: enhancing vision and beyond

It is estimated that about 43.3 million people are blind and 295 million with moderate to severe visual impairment worldwide in 2020 (Vision Loss Expert Group of the Global Burden of Disease Study, 2024). Retinal prostheses, a type of implantable electronic device, are designed to deliver electrical current to the retina to activate residual neurons for partial restoration of light perception in blind patients. Early generations of retinal prostheses involved implanting an electrode array on or under the retina, namely epiretinal and subretinal prostheses. These prostheses have limitations of low spatial resolution, off-target stimulation, low reliability, and mechanical and material stress to the retina. Nanoparticles (NP) that can transduce optical, acoustic, or magnetic fields into neural electrical responses have the potential to overcome these limitations and serve as next-generation retinal prostheses with the advantages of being less invasive and cell-type specific.

Stoddart et al. summarized NP transducers that can be activated by light for retinal neuromodulation in their review article. By conjugating NPs with versatile materials or ligands, retinal cells can be stimulated by light through different mechanisms, such as photothermal, photomechanical, photoacoustic, photovoltaic, and photochemical effects. Echoing this review, Rahmani and Eom reported an enhanced organic photovoltaic-based retinal prosthesis utilizing plasmonic NP to improve light-to-electricity conversion. This technology can significantly increase the efficiency of such devices while minimizing the physical footprint and energy requirements for patients with retinal degenerative diseases. Their computational analysis shows a 10% increase in current density for the photovoltaic cell with the developed structure containing K-AgNPs. Additionally, the simulation results indicate that the modeled device doubles the efficiency of a bare photovoltaic cell. As reviewed by Stoddart et al., these optical innovations offer promising tools that extend beyond traditional methods, providing new opportunities to restore visual functions.

## Optogenetics: a spectrum of therapeutic light

While NP-based retinal prostheses are still under investigation, optogenetics, which allows for precise control over neural activity through genetically encoded light-sensitive proteins, has shown effectiveness in partially restoring visual perception in retinitis pigmentosa patients (Sahel et al., 2021). The application of optogenetics in other neurological conditions is also under active exploration. One example is the research conducted by KC et al., which involved expressing Channelrhodopsin and Halorhodopsin in the trigeminal ganglion of a trigeminal neuralgia rat model. They found that inhibition of trigeminal ganglion through Halorhodopsin activation, rather than Channelrhodopsin, significantly reduced pain-related behavioral and electrophysiological signatures. These results suggest that optogenetics can effectively manage the pain in trigeminal neuralgia, offering a non-pharmacological alternative for chronic pain management.

Beyond its therapeutic potential in correcting abnormal neural activity in various neurological disorders, the specificity and reversibility of optogenetic modulation make it an invaluable tool for studying physiological and pathological neural circuits. In the research by Xiang et al., optogenetics was used to investigate the role of the orexinergic system in anesthesia arousal and pain modulation. Utilizing retrograde and anterograde labeling techniques, Xiang et al. delineated the pathways through which orexinergic neurons influence key brain regions involved in arousal. Furthermore, by optogenetically activating Channelrhodopsin expressing orexin neurons in the hypothalamus, robust arousal was elicited from light isoflurane anesthesia in mice. This insight opens new avenues for targeted interventions in anesthesia and analgesia, potentially leading to more effective and patient-specific treatment strategies. This synergistic integration of optogenetics with orexinergic research not only enhances our grasp of brain function but also paves the way for innovative therapeutic applications that could significantly improve the management of arousal and pain in clinical settings.

## Acoustic modulation: precise and non-invasive

Building on the principles of optogenetics while incorporating the non-invasive advantages of ultrasound, sonogenetics is emerging as a new neuromodulation approach that harnesses ultrasound to manipulate genetically modified cells. The review article by Wang et al. presents a comprehensive overview of ultrasound-responsive mediators, explores the molecular mechanisms underlying their response to ultrasound stimulation, and summarizes their applications in neuromodulation. The genetic tools are employed to facilitate the targeted expression of ultrasound-sensing proteins in specific cell types, thereby enhancing their sensitivity to ultrasound compared to unmodified cells. This approach allows for the selective activation of genetically modified target cells, without affecting naive cells, within regions exposed to focused ultrasound. Although still in its early stage, sonogenetics enables precise stimulation of specific brain regions or circuits without the need for surgical implants, which holds great promise for both clinical neuromodulation and basic neuroscience research.

## Integrating technologies: comprehensive solutions

The integration of optical, acoustic, and genetic neuromodulation technologies offers a holistic approach to both fundamental neuroscience research and the management of complex neurological conditions. This synergistic combination shows a prospect of more effective treatments, improved safety profiles, and deeper insights into brain function, potentially leading to novel therapeutic targets and better clinical outcomes.

## Prospects and challenges

Despite the immense potential of neuromodulation technologies, the journey from research to clinical application is fraught with significant challenges, including high development costs, lengthy regulatory processes, and the need for new clinical trial frameworks. Nonetheless, the future of neuromodulation is promising, with the potential to significantly transform the treatment landscape for neurological disorders.

## Conclusion: embracing a multidisciplinary future

Neuromodulation stands at the forefront of a multidisciplinary approach to treating neurological disorders, drawing on advancements in genetics, optics, and acoustics. The field not only promises to improve the lives of those affected by neurological disorders but also offers profound insights into brain function. Continued investment in research and thoughtful consideration of ethical issues will be essential for unlocking the full potential of these groundbreaking technologies.
